# Wavelength Modulation Characteristics of Metal Gratings on Si-Based Blocked-Impurity-Band (BIB) Terahertz Detectors

**DOI:** 10.3390/mi13050811

**Published:** 2022-05-23

**Authors:** Yulu Chen, Zuoru Dong, Yangzhou Zhou, Jiajia Tao, Wulin Tong, Yifei Wu, Wenhui Liu, Bingbing Wang, Xiaowan Dai, Xiaodong Wang

**Affiliations:** Shanghai Microwave Technology Research Institute, Shanghai 200063, China; chenyulu@50.sh.cn (Y.C.); feiyu3220180398@outlook.com (Y.Z.); 17112020036@fudan.edu.cn (J.T.); wptwl@live.com (W.T.); wyf773773@gmail.com (Y.W.); wenhui-liu@outlook.com (W.L.); wbb@mail.ustc.edu.cn (B.W.); monserdxw@outlook.com (X.D.)

**Keywords:** metal grating, Si-based blocked-impurity-band detector, wavelength modulation, multicolor detection

## Abstract

In this work, the wavelength selection characteristics of metal gratings on Si-based blocked-impurity-band (BIB) detectors in the terahertz band were studied by performing experiments and a finite difference time domain (FDTD) simulation. The transmission spectra of metal gratings with different periods on 130 μm intrinsic Si substrates were measured. When the metal grating period increased from 16 to 20 to 32 μm, the peak position of the spectrum moved from 21.71 to 24.50 to 36.59 μm, which is in good agreement with the FDTD simulation results. The structure with the period of 32 μm shows the best wavelength selective transmission characteristics. Then, the bare Si-based BIB devices and metal grating/Si-based BIB hybrid devices with different thicknesses of blocking layers of 2 and 5 μm were fabricated. By covering different periods of metal gratings for the devices with a thicker blocking layer of 2 μm, we obtained more effective wavelength selection characteristics and stronger response spectra enhancement ratios that were about 1.3, 2.4, or 1.9 times. This was mainly due to the localized optical field enhancement effect of the plasmons resonance in metal gratings, which decays exponentially in a vertical direction. Our results demonstrate a new approach for the Si-based BIB detector to realize multiband selective detection applications.

## 1. Introduction

The terahertz (THz) wave band (frequency range from 0.1 to 10 THz) contains the spectral fingerprint information of many celestial bodies and biological targets [1,2]. Therefore, THz detecting technology is regarded as a potential technical means for astronomical observation, medical diagnosis, nondestructive flaw detection, and so on. Many kinds of THz detecting technologies have been developed, such as the superconducting tunnel junction, the hot electron bolometer, and the blocked-impurity-band (BIB) detector. Among them, the Si-based BIB detector was widely studied for its high sensitivity, large-scale array, and wide spectra response of 4–30 μm [3,4]. It has already been used in many space exploration satellites, such as the Cosmic Background Explorer (COBE), the Spitzer Space Telescope (SST), and so on [5,6].

To further improve the detection efficiency and the integration of the detection system, multi-spectral simultaneous detection has been proposed and applied in visible light, infrared, ultraviolet, and other forms of waveband detection. However, traditional polychromatic detection is achieved by using mutually independent detection imaging devices and optical systems, such as filter wheels and spectrally separated tunable filters, which have large volume and high power consumption [7]. In the 1990s, the concept of multicolor focal plane array (FPA) was proposed; that is, a single integrated chip can realize simultaneous spectral detection of multiple bands, which enables the ability to tune each pixel individually [8]. Therefore, bicolor and multicolor FPA devices are becoming the important development direction for the next generation of detectors [9].

Recently, it was demonstrated that the selective transmittance of the metasurface, based on the concept of plasmons, can provide a good means for multiband FPA detection [10,11,12]. Jung et al. investigated a thin silver (Ag) layer perforated with an array of cross-patterned holes as a metasurface used in a multispectral microbolometer FPA, which exhibits excellent wavelength-selective narrowband absorption, allowing multispectral imaging in the long-wavelength infrared region [11]. Yakimov et al. indicated that the photoresponse intensity of the quantum dot mid-infrared photodetector can be enhanced about 15 times at a wavelength of 4.4 μm by covering an Au layer with two-dimensional subwavelength hole arrays, and that enhanced sensitivity arises from coupling the surface plasmon resonance and diffractive effect [13]. Based on this, a metal grating/Si-based BIB hybrid structure was designed and theoretically simulated in our previous work [14]. It was demonstrated that the absorption peak wavelength of the hybrid structure could be modulated from 24.46 to 36.53 μm by changing the metal grating periods from 8 to 32 μm. Moreover, the peak absorptivity of the hybrid structures was increased by more than 87.3%, compared with that of the bare Si-based BIB detector. Therefore, the metal gratings are an ideal and easily fabricated metasurface structure to realize selective resonant enhanced absorption and tunable spectral response for Si-based BIB detectors. However, there are few experimental results reported so far; reports exist only on the response spectra modulation phenomenon of the metal grating/BIB hybrid structures by the metal grating structures, operating voltages, and temperatures [15]. The underlying mechanism of metal gratings on the wavelength modulation characteristics needs to be further studied.

In this work, the transmission spectra of the metal grating/Si (130 μm) structures were studied by performing experiments and an FDTD simulation. The influence of individual metal grating structures with different periods on the wavelength selective transmission characteristics of incident light was deeply investigated. To study the effect of the metal grating on the Si-based BIB detector further, Si-based BIB detectors and metal grating/Si-based BIB detectors with different thicknesses of blocking layers (BLs) were fabricated. It was found that the response spectra of the devices with a 2 μm-thick BL show better wavelength selection and amplification characteristics compared to the BLs with a thickness of 5 μm. This is based on the strong distance dependence of the optical field’s localized effect on the plasmons in metal gratings. This work provides a scientific basis for the multiband detection of metal grating/Si-based BIB-integrated detectors.

## 2. Materials and Methods

### 2.1. Device Fabrication and Measurement Processes

**Figure 1:**
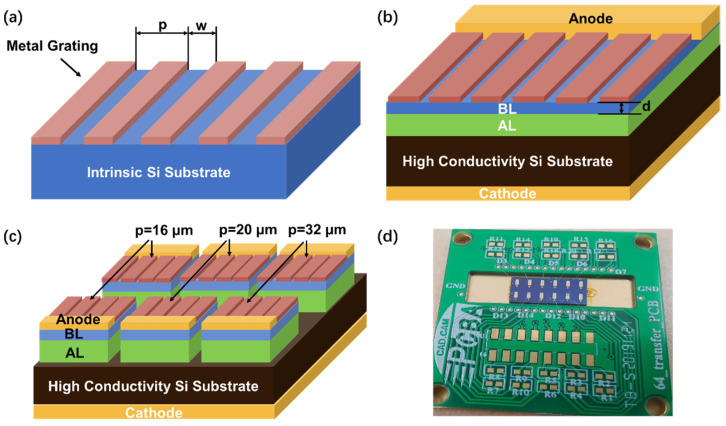
(**a**) Structure diagram of metal grating/Si (130μm), in which p is the metal grating period; w is the interval width between the metal strips; and the duty ratio, (p−w)/p, is fixed at 3/4. (**b**) Structure diagram of metal grating/Si-based BIB detector, in which d is the thickness of the BL. (**c**) Structure diagram and (**d**) photo of 3 × 2 array multicolor metal grating/Si-based BIB detector.

To directly study the modulation effect of metal grating and exclude the influence of the response peak of the BIB detector, metal grating/Si-intrinsic substrate hybrid structures were designed and fabricated, since there is no corresponding energy level in the intrinsic Si to absorb the incident light in the wavelength range of 4–50 μm. The structure of the metal grating/Si substrate hybrid structure is shown in Figure 1a. The 500 μm-thick Si substrate was thinned to 130 μm using a horizontal rotary grinding machine (HRG-150) followed by chemical mechanical polishing (AP-380F), and then divided into three pieces. Considering the response spectrum of the bare Si-based BIB detector and manufacturing limitations of small-period metal grating [14,15], the metal gratings with different periods of 16, 20, and 32 μm and a duty ratio of 3/4 were deposited on the pieces by photolithography and electron beam evaporation with Ti (100 nm)/Al (4900 nm) materials, respectively. Al is selected for metal grating because of its mature preparation process and good compatibility with the devices used.

The structure of the metal grating/Si-based BIB detector is shown in Figure 1b, including the Si substrate, Si absorption layer (AL), Si BL, cathode, anode, and metal grating structure. Under the incident light irradiation, the AL fully absorbs the incident light and the BL suppresses the dark current. The fabrication process is as follows: (1) a highly conductive Si substrate with resistivity of 0.001~0.005 Ω·cm was selected here; (2) a 10 μm-thick phosphorus (P)-doped absorption layer (AL) was deposited on the Si substrate at 1100 °C by chemical vapor deposition (CVD) equipment with the gas sources of SiHCl_3_ and PH_3_, where the doping P concentration reaches 8 × 10^17^ cm^−3^; (3) then the intrinsic BL was grown on the AL with a thickness of 2 or 5 μm at 1100 °C by CVD with the gas source of SiHCl_3_; (4) before metal electrodes were deposited, the electrode contact layers for anode and cathode were formed by the implantation of P atoms, followed by a rapid annealing process with a temperature value of 1000 °C for 15 s to activate the implanted P atoms and repair the lattice damage; (5) next, the anode and the cathode were deposited by electron beam evaporation with a Ti(20 nm)/Al(120 nm)/Ni(20 nm)/Au(200 nm) layer, followed by 400 °C annealing for 30 min in N_2_ atmosphere to form ohmic contact; (6) finally, the metal gratings were fabricated by photolithography and electron beam evaporation as the same parameters in metal grating/Si structures. Based on this, a 3 × 2 array multicolor metal grating/Si-based BIB detector with a pixel size of 1 × 1 mm^2^ was fabricated. The periods of metal grating on one row of pixels were 16, 20, and 32 μm, respectively, as shown in Figure 1c,d.

The transmission spectra (i.e., transmissivity) of the metal grating/Si (130 μm) hybrid structures and the spectral response of the metal grating/Si-based BIB detectors were measured at 4 K by a Bruker VERTEX 80V Fourier transform infrared (FTIR) spectrometer. The normalized transmissivity spectrum of the metal grating/Si was obtained by dividing the transmissivity spectrum by its peak intensity.

### 2.2. Device Simulation

Lumerical FDTD solution (version: 8.15.736) was used to simulate the optical properties of the metal grating/Si (130 μm) and metal grating/Si-based BIB hybrid structure detector, especially transmission spectra and optical field distributions. The simulation models of the devices consisted of geometric parameters, complex refractive indexes, dielectric constants, metal conductivity models, IR and THz wave sources, the monitors for reflection and transmission record, boundary conditions, and so on. The measured material parameters and actual device parameters, such as the complex refractive index, dielectric constant, and geometric parameters, were used in the simulation. The THz source was set as a plane wave and the angel of the THz wave was zero degrees. The boundary condition was periodic. The locations of the monitors are described in detail below.

## 3. Results and Discussion

**Figure 2:**
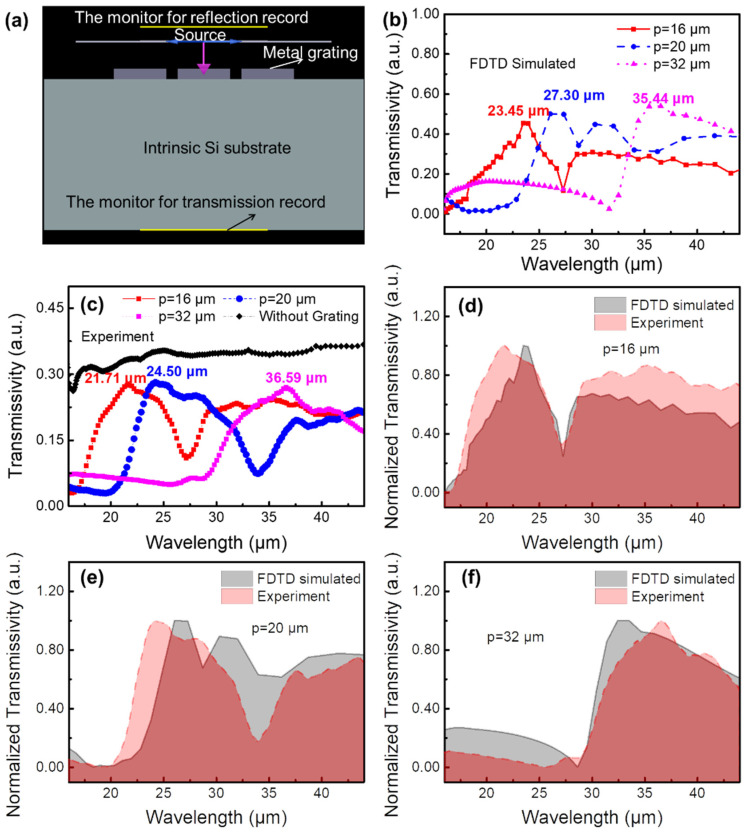
(**a**) FDTD model, (**b**) the simulated transmission spectra of the metal grating/Si structure, in which the periods of the metal gratings are set as 16, 20, and 32 μm, respectively. The light source, monitors, and the device structure are marked in (**a**). (**c**) The experimental transmission spectra of the bare Si (130 μm) substrates and the Si (130 μm) substrates with 16, 20, and 32 μm periods of metal grating, respectively. (**d**–**f**) One-by-one comparison of the normalized FDTD simulation and experimental transmission spectrum results of the three hybrid structures with different periods of metal gratings.

The FDTD simulation model of the metal grating/Si hybrid structure is shown in Figure 2a. The parameters used in the model are consistent with the experiment parameters. The wavelength of incident light is set in the range of 4–50 μm. The periods of the metal gratings are set as 16, 20, and 32 μm, respectively. Figure 2b shows the simulated transmission spectra of the hybrid structures with different periods of metal gratings. The peak position of the spectrum moves from 23.45 to 27.30 to 35.44 μm, when the metal grating period increases from 16 to 20 to 32 μm. The experimental results in Figure 2c demonstrate the same tendency, whereby the peak position changes from 21.71 to 24.50 to 36.59 μm with the increasing grating period. According to the Drude theory [14,16,17], the plasmon resonance frequency of metal gratings, $\omega_{p}$, can be calculated by the formula:

(1) $$\omega_{p}^{2}=\frac{n_{eff}e^{2}}{\varepsilon_{0}m_{eff}}=\frac{4c^{2}}{pw^{\;}},$$

where $n_{eff}$ is the effective electron density, e is the electron charge, $\varepsilon_{0}$ is the vacuum dielectric constant, and $m_{eff}$ is the electron effective mass. Thus, the relationship between the resonance wavelength, $\lambda_{p}$, and the parameters of metal grating, p and w, can easily be deduced as follows:

(2) $$\lambda_{p}=\frac{2\pi c}{\omega_{p}}=\sqrt{\pi^{2}pw}$$

Therefore, the peak position of the transmission spectra red shifts when the period and the interval of the metal grating increase. In addition, the transmissivity of all metal grating/Si structures being lower than that of the Si substrate (black line in Figure 2c) means higher absorption characteristics, which are essential for improving device performance. Figure 2d–f show the comparison of the normalized FDTD simulation and experimental transmission spectrum results of the three hybrid structures with different periods of metal gratings, respectively. The one-by-one comparison shows that the experimental results are basically consistent with the theoretical ones, which proves the accuracy of the simulation model used here. The fine deviations are mainly caused by light scattering and refraction in the transmissivity measurement process [15]. The structure with the period of 32 μm shows the best wavelength-selective transmission characteristic for a peak with the full width at a half-maximum (FWHM) of about 13.8 μm.

**Figure 3:**
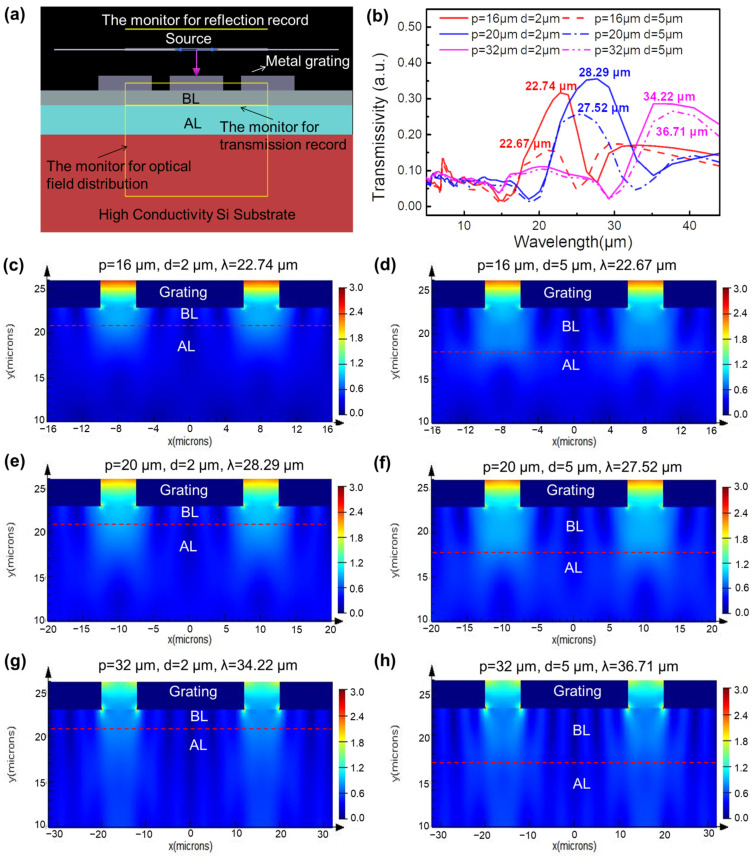
(**a**) FDTD model, (**b**) the simulated transmission spectra of the metal grating/Si-based BIB detector, in which the periods of the metal grating are set as 16, 20, and 32 μm, and the thicknesses of the BL are 2 and 5 μm, respectively. The light source, monitors, and the device structure are marked in (**a**). (**c**–**h**) The optical field distribution of different devices, in which the red dashed lines mark the interface between the AL and the BL.

Figure 3a shows the FDTD model of the metal grating/Si-based BIB detector. The thicknesses of the BL and the AL are set as 2 (or 5) and 10 μm, respectively. There are two monitors, one below the BL and the other covering the entire BL and AL, which monitor the transmission of light through the BL (e.g., the incident light entering the AL) and the optical field distribution inside the device, respectively. In Figure 3b, for the devices with BLs of the same thickness, the peak position of the transmission spectrum red shifts as the metal grating period increases, which is approximately the same as that of the corresponding metal grating/Si (130 μm). However, for the devices with the same period of metal grating, the incident light entering the AL for the device with the 2 μm-thick BL is higher than that of the device with the 5 μm-thick BL. It cannot be caused by the absorption of the BL because there are no corresponding energy levels in the intrinsic Si BL to absorb the incident light in the wavelength range of 4–50 μm. Figure 3c–h show the simulated optical field distributions in the hybrid structure devices, in which the red dashed lines mark the interface between the AL and the BL. It can be found that for the devices with the same period of metal grating, the optical field intensity reaching the AL in the device with the 2 μm-thick BL is higher than that of the device with the 5 μm-thick BL. This is mainly due to the fact that the optical field localized effect caused by the plasmon resonance reaches its maximum at the edge of the metal grating, and then decreases exponentially down into the BL. When the AL is far away from the metal grating layer, the localized optical field enhancement effect attenuates accordingly.

**Figure 4:**
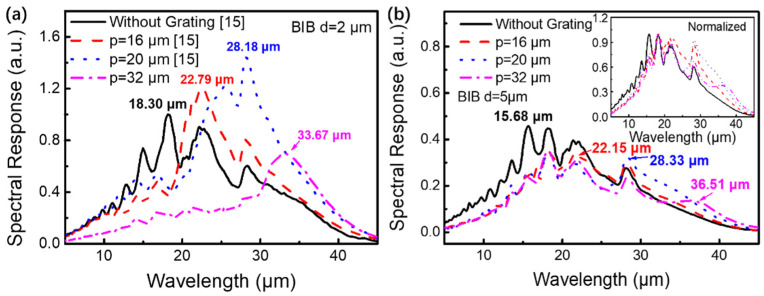
Experimental response spectra of the Si-based BIB detectors covering with and without 16, 20, and 32 μm periods of metal gratings, in which the thicknesses of the BL are (**a**) 2 μm and (**b**) 5 μm, respectively. The black, red, and blue spectra curves in (**a**) are reprinted with permission from Ref. [15]. 2022, Applied Physics Letters. The inset image in (**b**) is the normalized response spectra.

The response spectra of the bare Si-based BIB detectors and the metal grating/Si-based BIB detectors with BLs of different thicknesses (2 and 5 μm) were measured at 4 K, as shown in Figure 4a,b, respectively. The cut-off wavelengths of the bare Si-based BIB detectors (the black curves) are approximately 40 μm (~31 meV), which is close to the activation energy of the P impurity band in the Si matrix [18]. For the bare BIB detector with a 2 μm BL, a main peak and four shoulder peaks located at 18.30, 12.81, 15.00, 22.30, and and 28.33 μm can be observed. These peaks result from the interference of the incident light at different interfaces [15]. When the Si-based BIB detector was covered by a 16, 20, or 32 μm period metal grating, the main peak shifted from 18.30 to 22.79, 28.18, and 33.67 μm, which is consistent with the simulation transmission spectra in Figure 3b. Notably, after covering the 16, 20, or 32 μm period metal grating structures, the intensity of the main peak increased by 1.3, 2.4, or 1.9 times, respectively, due to the localized optical field enhancement effect of the plasmon resonance. When the thickness of the BL increased to 5 μm, the response spectrum intensity of the Si-based BIB detector decreased due to the enhanced resistance of the BL, as shown in Figure 4b. The main response peak position of the bare BIB detector (the black curve in Figure 4b) was located at 15.68 μm. When the device was covered by a metal grating with a 16, 20, or 32 μm period, the intensity of the response spectrum increased by 0.8, 1.2, or 1.8 times, and the main peak position shifted from 15.68 to 22.15, 28.33, or 36.51 μm, respectively, which is in line with the optical field distribution results in Figure 3. Obviously, the metal grating/Si-based BIB hybrid detector with the 2 μm-thick BL displays more effective wavelength selection characteristics and a stronger response spectra enhancement ratio, suggesting it to be more conducive to the preparation of the multicolor terahertz detector. To avoid the large dark current and noise, and also to ensure a low impurity concentration in the BL, the thickness of the BL cannot be further reduced below 2 μm.

## 4. Conclusions

In this work, both theoretical and experimental methods were used to investigate the wavelength modulation performances of the metal gratings on the Si-based BIB detectors. The Si (130 μm) substrates covered by 16, 20, and 32 μm period metal gratings were fabricated and then the transmission spectra were measured at 4 K accordingly. The peak position of the spectrum moves from 21.71 to 24.50 to 36.59 μm with the increasing grating period. In addition, the bare Si-based BIB devices and metal grating/Si-based BIB hybrid devices with 2 and 5 μm-thick BLs were fabricated. It was found that the devices with 2 μm-thick BLs display more effective wavelength selection characteristics and stronger response spectra enhancement ratios of about 1.3, 2.4, or 1.9 times compared to those with 5 μm-thick BLs. This is attributed to the localized optical field enhancement effect of the plasmon resonance in different periods of metal gratings. A 3 × 2 array metal grating/Si-based BIB detector was fabricated with different periods, which provides a good foundation for the multicolor terahertz detector applications.

## Data Availability

The data that support the findings of this study are available from the corresponding authors upon reasonable request.
